# Troxerutin suppress inflammation response and oxidative stress in jellyfish dermatitis by activating Nrf2/HO-1 signaling pathway

**DOI:** 10.3389/fimmu.2024.1369849

**Published:** 2024-05-08

**Authors:** Ran Liu, Yulian Wang, Wenhao Kuai, Wenting Li, Zengfa Wang, Liang Xiao, Jianhua Wu

**Affiliations:** ^1^ Department of Dermatology, The First Affiliated Hospital of Naval Medical University, Navy Medical University, Shanghai, China; ^2^ Faculty of Naval Medicine, Naval Medical University, Shanghai, China; ^3^ College of Traditional Chinese Medicine, Jilin Agricultural University, Changchun, China

**Keywords:** jellyfish dermatitis, inflammation response, oxidative stress, troxerutin, MAPK signaling pathway, erythroid 2-related factor 2 signaling pathway

## Abstract

**Background:**

*Stomolophus meleagris* envenomation causes severe cutaneous symptoms known as jellyfish dermatitis. The potential molecule mechanisms and treatment efficiency of dermatitis remain elusive because of the complicated venom components. The biological activity and molecular regulation mechanism of Troxerutin (TRX) was firstly examined as a potential treatment for jellyfish dermatitis.

**Methods:**

We examined the inhibit effects of the TRX on tentacle extract (TE) obtained from *S. meleagris in vivo* and *in vitro* using the mice paw swelling models and corresponding assays for Enzyme-Linked Immunosorbent Assay (ELISA) Analysis, cell counting kit-8 assay, flow cytometry, respectively. The mechanism of TRX on HaCaT cells probed the altered activity of relevant signaling pathways by RNA sequencing and verified by RT-qPCR, Western blot to further confirm protective effects of TRX against the inflammation and oxidative damage caused by TE.

**Results:**

TE significantly induced the mice paw skin toxicity and accumulation of inflammatory cytokines and reactive oxygen species in vivo and vitro. Moreover, a robust increase in the phosphorylation of mitogen-activated protein kinase (MAPKs) and nuclear factor-kappa B (NF-κB) signaling pathways was observed. While, the acute cutaneous inflammation and oxidative stress induced by TE were significantly ameliorated by TRX treatment. Notablly, TRX suppressed the phosphorylation of MAPK and NF-κB by initiating the nuclear factor erythroid 2-related factor 2 signaling pathway, which result in decreasing inflammatory cytokine release.

**Conclusion:**

TRX inhibits the major signaling pathway responsible for inducing inflammatory and oxidative damage of jellyfish dermatitis, offering a novel therapy in clinical applications.

## 1 Introduction

Jellyfish, an invertebrate organism, is among the world’s most toxic creatures. Jellyfish stings have become a threat to human activity owing to their explosive growth in recent years (1). Stomolophus meleagris (S. meleagris) is the most widely envenomed jellyfish species that blooms that spread in China. Korea, and Japan. The nematocysts released from S. meleagris, triggered by contact, can deliver strong and fast-acting venom into the epidermis, causing a range of clinical symptoms, from moderate discomfort to severe pain with necrosis and scarring. Dermatitis is a common condition that causes severe local cutaneous discomfort and is characterized by extreme pain and tissue damage, which greatly distress long-term fishery practitioners, tourists, and clinical researchers (2, 3). However, the molecular processes toward these envenoming effects are unclear. To minimize inflammation and provide widespread neutralization against S. meleagris venom, various treatments are utilized for the initial management of jellyfish dermatitis to decrease inflammatory responses and provide widespread neutralization against scorching; nevertheless, their efficacy is restricted. Notably, considerable debate and contention exist regarding the efficacy of existing treatments, with some potentially exacerbating symptoms (4, 5). Therefore, it is crucial to analyze the mechanism of skin inflammation induced by S. meleagris venom to develop effective clinical medicines.

Inflammation is considered a contributing factor to the course of jellyfish stings (3, 6). Proteomic and transcriptome analyses have shown that jellyfish venom is a mix of antigens and various proteases (7–9). Proteins and peptides in venom may act as potential antigens to increase the immune system’s release of inflammatory cytokines, leading to persistent dermatitis (10, 11). Bioactive compounds enter the microvascular beds of the skin, causing anaphylactic shock and multiorgan dysfunction (12, 13). Nevertheless, oxidative stress can be accompanied by inflammatory responses and increase the risk of numerous illnesses. The overproduction of reactive oxygen species (ROS) within cells and exhaustion of antioxidant defenses are believed to be the most effective pro-inflammatory signaling pathways that cause cell necrosis (14). Researches has shown the injection of crude Pelegia noctiluca envenomation elicited an inflammatory response and apoptosis, which were correlated with the plasma concentrations of ROS and nitric oxide (NO) (15). Oxidative stress from major indicators of mouse serum lipid peroxidation and the accumulation of intercellular ROS may be additional pathways for the effects of jellyfish venom (16, 17). According to recent findings, skin oxidative damage and inflammation induced by Nemopilema nomurai can be mitigated through a class of natural antioxidant algal-derived polysaccharides by inhibiting mitogen-activated protein kinases (MAPK) and nuclear factor-kappa B (NF-κB) pathways (18). ROS accumulation between cells leads to activate the MAPK signaling pathway, which further aggravated inflammation response (19).. However, few studies about S. meleagris TE induced inflammatory response and oxidative stress in MAPK pathway studies. Accordingly, research on inflammatory mediators and oxidative stress-related signaling pathways should be considered in the theory of inflammatory skin disorders (8, 9).

Troxrutin (TRX) is a naturally occurring flavonoid that has undergone hydroxymethylation and contains an range of biological characteristics, including antioxidant, anti-inflammatory, immune-stimulating, and anticancer properties. It has been clinically recognized the anti-inflammatory effect of TRX was proved by performing extensive cellular and animal experiments to treat various diseases (20–22). The malondialdehyde (MDA) levels can be significantly reduced in rat hippocampal tissue by TRX application and combating the apoptosis of hippocampal neurons by enhancing the activities of Superoxide Dismutase (SOD) glutathione peroxidase (GPx) (23). By down-regulating the expression of inflammatory factor interleukin-6 (IL-6), tumor necrosis factor-α (TNF-α), and cyclooxygenase (COX-2), TRX prevented the NF-kappaB and MAPK signal pathway from being activated, which inhibited the inflammatory response of advanced glycation end products (AGEs) in a mouse osteoarthritis injury model (24). Furthermore, troxerutin protected HacaTcells from UVB-induced decrease in cell growth through regulated miRNA function and suppressed apoptosis (25). In addition, TRX can reduce Edema and capillary hydrostatic pressure through hyaluronidase and histamine syntheses by TRX application, resulting in the protection of vascular endothelial cells and the effective improvement of microcirculation (26). Notably, Histamine, 5-hydroxytryptamine, and kinin in venom of jellyfish can operate to contract and spasm vascular smooth muscle and increase the permeability of nearby blood vessels, causing severe discomfort and localized skin congestion and edema (7, 27). Nevertheless, there are no reliable remedies to relieve venom-mediated skin edema and pain due to increased skin capillary permeability. Due to these biological properties of TRX mentioned above, we plausibly hypothesized that TRX may protect skin HacaT cell against jellyfish dermatitis development and further studies of TRX’s antagonistic effects are needed.

In the study, the toxic effects of the tentacle extract (TE) obtained from S. meleagris venom were investigated *in vivo* and vitro. Furthermore, TRX inhibits the inflammatory factors of TE-induced and enhances the activity of antioxidant enzymes. The beneficial function of TRX, which in mitigating skin inflammation responses and oxidative stress in jellyfish dermatitis, were further identified by related oxidation signaling pathway and RNA-seq analysis and to explore the possible active molecular mechanisms. This study may provide an innovative strategy for further research on the therapy of jellyfish stings.

## 2 Materials and methods

### 2.1 Jellyfish collection and tentacle isolation

Live *S. meleagris* specimens were collected from Bohai Bay, Dalian City, Liaoning Province, China. Tentacles were immediately removed from the freshly captured jellyfish and frozen on dry ice. samples were brought to the lab and kept at -80°C.

### 2.2 Jellyfish venom extraction and protein concentration determination

Nematocyst venom was extracted using established methods (28). Frozen *S. meleagris* tentacles were frozen and then thawed at 4°C in a beaker. After 72 h, the defrosted tentacle extract was continually churned in a magnetic mixer at 4°C until no discernible tissue mass remained. The autolysis extract was filtered twice through a 200-mesh screen and collected after being centrifuged for 15 minutes at 1000 ×g at 4°C. The venom was then placed in a dialysis bag, where it was agitated by magnetic stirrers overnight at 4°C in 1× phosphate-buffered saline (PBS) (pH 7.4). Following dialysis, *S. meleagris* venom was extracted, divided into 15 ml centrifuge tubes, and kept for later use in a freezer at -80°C. The Bicinchoninic Acid Assay (BCA) protein concentration detection kit (Beyotime, China) was used to calculate the amount of *S. meleagris* TE protein. The measured protein concentration can serve as a proxy for jellyfish venom protein content in future studies. The final TE concentration used in this study was 2.5 mg/mL.

### 2.3 Mice paw swelling models

#### 2.3.1 Animal maintenance

In order to mitigate the possible impact of hormone fluctuations and additional reasons for differences related to sex, only male mice were used in this investigation. Improved Castle Road (ICR) mice (20 ± 2 g, male) were purchased from the Navy Medical University Experiment Animal Center. The animals had been raised in a facility with controlled temperatures and regular day and night cycles (12 h light/dark cycle). The mice were able to obtain water and food. All animal experiments were approved by the Shanghai Changhai Hospital Committee on the Ethics of Animal Experiments at the Chinese Naval Medical University. (Ethical approval number: CHEC (A.E)2022-003).

#### 2.3.2 Dermal toxicity in animals

To assess the dermal toxicity of S. meleagris TE, mice were subcutaneously injected with different doses (5, 15, 50 ug) dissolved in normal saline (NS). Mice were randomly assigned to four groups (six mice in each group), and a subcutaneous injection of TE was administered to the mice’s plantar area skin. The erythema and edema symptoms of the mouse paws in each group and the percentage of swelling of the mouse paw were scored according to the skin irritation reaction criteria and the maximum dorsoventral thickness of the left paw at different periods. The percentage of swelling was calculated using the formula: Swelling percentage = paw thickness at each stage after injection - paw thickness before injection)/paw thickness before injection * 100%. The swelling percentage of the mouse paws was plotted with time (h) as the horizontal coordinate and the swelling percentage of the paw (%) as the vertical coordinate. Furthermore, after the mice’s cervical dislocation death, blood samples were taken to measure the serum levels of inflammatory factors. Similarly, to examine how TRX affects TE-induced cutaneous toxicity, mouse paws were treated with a mixture of venom and TRX (45 mg/kg), which was premixed for 30 min to maintain the final concentration of venom at 1 mg/mL. TRX’s inhibitory effect on paw erythema, edema, and serum inflammatory factors was measured as described above. Mouse paw skin tissue was subcutaneously injected with TRX to show that the body was not affected by inflammation. The negative control group consisted of animals that received only vehicle (NS).

### 2.4 Cell culture and troxerutin treatment

The HaCaT cells (Fuheng Biological Co., Ltd., Shanghai, China), identified by STR technology, were raised in Dulbecco’s Modified Eagle Medium (DMEM) medium maintained at 37°C in a humidified atmosphere with 5% CO2 and 10% fetal bovine serum added (Lonsrea A511-001, Australian) and 1% penicillin/streptomycin mixture (Grand Island, NY, USA). Troxerutin (TRX) (purity, ≥ 97%; molecular weight, 742.67; formula, C33H42O19) was purchased from Shanghai Macklin Biochemical Co., Ltd (Shanghai, China). At a concentration of 10 mM (pH 7.4), TRX was created in PBS and kept at -20°C.

### 2.5 Cell viability

The HaCaT cells were plated in 96-well plates (1×104 cells/well) after being incubated for 24 hours. Cells were treated with TE at varying concentrations (2, 6, 12, 20, 60, 120, 200 μg/mL) for 2 h, and the CCK-8 assay (TopScience, Shanghai, China) was used to monitor cell viability. To detect the antagonistic effects of TRX, TE and TRX (20, 50, 200, 500, 1000 μm) mixed solutions were incubated with HaCaT cells for 2 h under the stimulus of a fixed venom concentration (10 μg/mL). Absorbance was determined at 450 nm following the addition of the CCK-8 reagent.

### 2.6 Analysis of intracellular ROS production

After being cultivated on 12-well plates for 24 h at a density of 1×105 cells/well, the cells were stimulated with 10, 30, and 60 μg/mL of TE. After 2 h of incubation, the intracellular ROS generation was assessed according to the reactive oxygen species ROS kit (Beyotime, China). The fluorescent probe DCFH-DA was diluted in serum-free medium (DMEM) to a final concentration of 10 μM/L. Cells were collected and suspended in diluted DCFH-DA, incubated at 37°C for 20 minutes with appropriate inversion and mixing, so that the probe and cells were in full contact with each other. The ratio of fluorescence intensity was performed using a CytoFLEX flow cytometer (Beckman Coulter, Brea, CA, USA) with emission and excitation wavelengths of 488 and 525 nm, respectively. In addition, 50µM DHE fluorescent probe staining solution was prepared and mixed with the treated cells, which were detected by fluorescence microscope (Rockford, IL, USA) under excitation wavelength of 510 nm and 600 nm emission wavelength. The imageJ was used to analyze the quantitative of relative fluorescence intensity. For drug intervention, HaCaT cells were treated with mixed solutions containing TRX (1 mM) and TE (10 μg/mL) and incubated for 2 h, similar to the previous detection method to test TRX inhibition on TE-induced oxidant stress.

### 2.7 Enzyme-linked immunosorbent assay analysis

The survival rate of HaCaT cells was approximately 80% to 90%, with a venom concentration of 10 μg/mL as the modeling concentration. After 24 h incubation, cells were treated with TE, and the supernatants were collected using a replaceable centrifuge rotor (3000 rpm; Thermo Scientific™, X1 Pro, USA). The protein expression levels of inflammatory factors interleukin-6, interleukin-8 (IL-8), TNF-α, interleukin-1β (IL-1β), and oxidation factor of MDA and ROS were examined using 96-well ELISA kits(Institute of Biological Engineering of Nanjing Jiancheng, China). Add 50 μL of standard and 10 μL of treated cell supernatant, respectively. Filled with horseradish peroxidase (HRP)-labeled antibody incubated at 37°C for 60 min. Discard the supernatant and incubated with 50 μL of substrate at 37°C for 15 min. Measure the OD value of each well at 450 nm. The concentration of the standard and the corresponding OD value were used as the horizontal and vertical coordinates, respectively. The protein concentration was calculated according to the equation of the linear curve of the standard. In addition, blood from the orbital veins of the animal model group was collected and left at 37° for 2 h. It was analyzed for relevant serum indicators of inflammation and oxidative stress in compliance with the provided similar method by the Elisa kit. For drug intervention, HaCaT cells were treated with mixed solutions containing TRX (1 mM) and TE (10 μg/mL) and incubated for 2 h, similar to the previous detection method to detect TRX inhibition.

### 2.8 Bioinformatics analysis

Following treatment in triplicate with 10 μg/mL *S. meleagris* venom or control (DMEM) for 2 h, For RNA sequencing analysis, HaCaT cells were gathered and kept at -80°C. Genes with differential expression (DEGs) were chosen. The Kyoto Encyclopedia of Genes and Genomes enrichment analysis and Gene Ontology studies were carried out with R software (LC-Bio Technology Co., Ltd., Hangzhou, China).

### 2.9 Reverse transcription polymerase chain reaction analysis

Before TE (10 μg/mL) stimulation, the cells were cultured for 24 h and treated with different doses of TRX. Cells were collected and homogenized using a RAN extraction kit (Beijing, Shanghai, China). The sample was dissolved in Diethylpyrocarbonate (DEPC) water after discarding the supernatant to obtain total RNA, total RNA was combined with a TaqMan MicroRNA Reverse Transcription Kit (Toyobo, Osaka, Japan) to obtain cDNA. SYBR-green (TargetMol, Shanghai, China) and particular gene primers were used to perform RT-qPCR on cDNA, with 30 PCR cycles: 5 min of denaturation at 94°C, 20 s of annealing at 60°C, and 30 min of extension at 72°C.

### 2.10 Western blot analysis

To prepare for TE (10 μg/mL) stimulation, 1×10**^6^** cells/dish were planted in 6 cm culture dishes, treated with TE or 1 mM TRX and TE mixture, left for 24 h. The protein extraction reagent SDS (LC-Bio Technology Co., Ltd., Hangzhou, China) was used to extract proteins from the cells and tissues. After estimating protein concentrations using the Protein Assay Kit for BCA, 10% polyacrylamide gels were used to electrophorese 10μg of total protein from each lane. Polyvinylidene difluoride membranes (Cytiva, Germany) were lined with the resolved protein bands and blocked with a buffer without protein for rapid blocking for 2 h (Ya Mei, Shanghai, China). Subsequently, the membranes were sequentially incubated with different monoclonal primary antibodies (1:1000) overnight at 4°C. After incubating with the primary antibody, the secondary antibody was washed with TBST and allowed to sit at 27°C for 1 h. An imaging device and enhanced chemiluminescence (ECL) chemicals were used to observe protein bands.

### 2.11 Statistical analysis

GraphPad Prism 7 (GraphPad Software Inc., La Jolla, CA, USA) and Origin 2018 were used for statistical analyses. Every test was carried out in triplicate, and the results are shown as means ± SD. This study compared groups in numerous ways using a one-way or two-way analysis of variance and compared two groups using Student’s t-test. The related figures were marked with an asterisk (*) to indicate statistical significance, with a p-value of less than 0.05.

## 3 Results

### 3.1 TE increased skin toxicity and inflammation *in vivo* and *in vitro*

**Figure 1:**
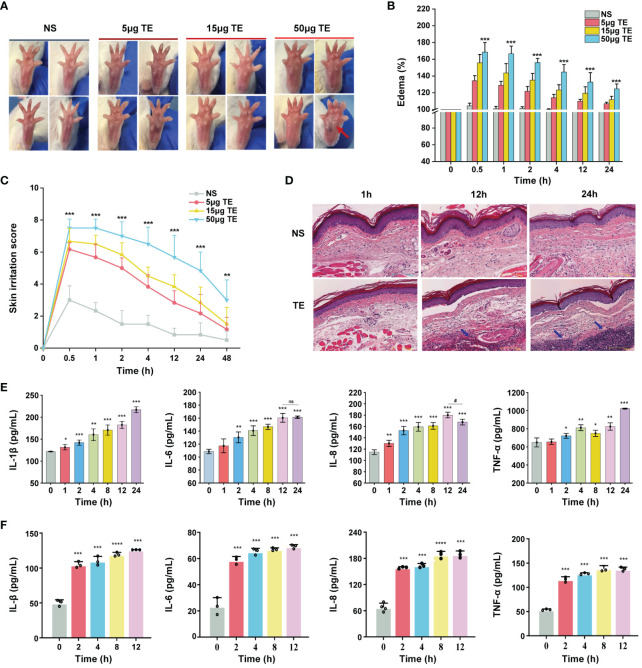
Toxic effect and inflammatory responses of the tentacle extract (TE) from *Stomolophus meleagris (S. meleagris) in vivo* and vitro. **(A)** TE induced the toxicity effect in mice paws at different times. **(B)** skin swelling percentage and **(C)** irritation score in mice paws. **(D)** section on dermal toxicity-related pathology. The histopathological slice’s scale bars are 100 μm in length. Blue arrowheads indicate the presence of inflammatory cells in subcutaneous tissue. **(E)**The levels of inflammatory factor protein expression, including interleukin-1β (IL-1β), interleukin-6 (IL-6), interleukin-8 (IL-8), and tumor necrosis factor-α (TNF-a) with TE (50 μg) in the blood of mice. and **(F)** in HaCaT cell with TE (10 μg/mL). n = 4 experiments/group. Three independent assessments of the determinant (n = 3, mean ± SE) verified the outcomes. A significant difference is shown by 4error bars with distinct letters (p < 0.05). *p < 0.05, # p < 0.05, **p < 0.01, ***p < 0.001, ****p < 0.0001, ns>0.05.

To determine whether TE exerts toxic effects on the epidermal skin, an animal model was used to investigate dermal toxicity. TE was administered subcutaneously at 5, 15, and 50 μg consecutively into the left paw of the mice. As predicted, the paws of the mice in the TE group exhibited a significantly higher inflammatory response. In addition to significant edema of the soles and toes, pronounced swelling and erythema were observed with venom stimulation in a dose-dependent manner (**Figure 1A**). Further, the percentage of mouse paw swelling increased in a dose-dependent manner, reaching its highest value at 0.5–1 h and then with a slight decline. Notably, the swelling percentage increased by more than 60% with TE at a concentration of 50 μg compared with that in control before injection (**Figure 1B**). Therefore, we determined TE concentration with 50 μg for further experiment. Similarly, skin irritation scores peaked within 1 h and decreased significantly 2 h after injection, which was consistent with the paw swelling percentage (**Figure 1C**). Histopathological investigations also revealed a considerable variation in mouse skin tissue inflammation caused by TE (**Figure 1D**). The venom group showed a propensity for edema and necrosis, evidenced by the significant thickness of the dermis at 1 h. Moreover, the number of inflammatory cells in the cutaneous tissue of the mice increased noticeably at 12 and 24 h, indicating that TE elicited an inflammatory response in the organism. Inflammatory cells penetrating the skin is mainly mediated by an inflammatory response (29). After the injection of TE into the mouse paws, the levels of inflammatory indicators in the blood of the mice were assessed for TE-induced damage from inflammation. Notably, acute inflammatory reactions were triggered, resulting in significant elevations in the inflammatory agents’ protein expression levels, such as IL-1β, IL-6, IL-8, and TNF-a (**Figure 1E**).

Next, we verified the effects of TE on human keratinocyte HaCaT cells. First, we evaluated the cytotoxic effect on HaCaT cells treated at various TE concentrations (0, 2, 6, 12, 20, 60, 120, and 200 μg/mL). The results showed that TE significantly reduced cell viability at concentrations ranging from 20–200 μg/mL compared with untreated cells (**Supplementary Figure S1**). Therefore, we chose TE with a concentration of 10 μg/mL for the subsequent experiment. Additionally, the application of TE to HaCaT cells led to a notable increase in inflammatory damage and marked upregulation of inflammatory cytokine expression, suggesting that TE can trigger an inflammatory response (**Figure 1F**).

### 3.2 TE-induced production of serum and intracellular ROS

**Figure 2:**
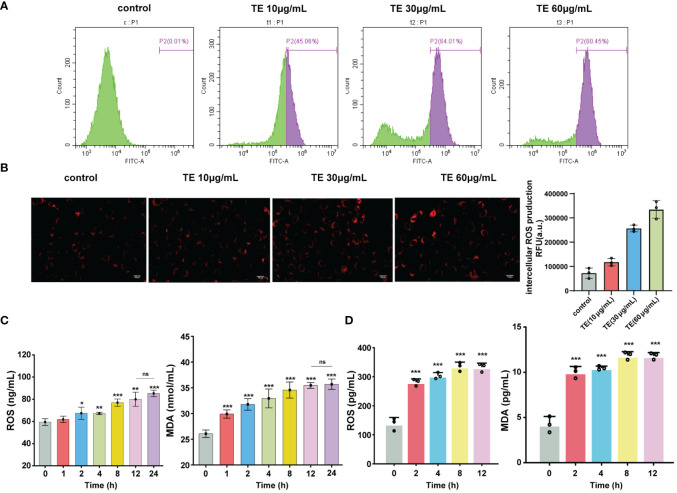
Oxidant stress stimulated by TE *in vivo* and *in vitro*. **(A)** Intracellular ROS generation, analysis of ROS level with TE (10, 30, and 60 μg/mL) by cytometer, and **(B)** fluorescence microscopy. **(C)** Reactive oxygen species (ROS) and malondialdehyde (MDA) are markers of oxidative stress, as seen in the blood of mice treated with TE and **(D)** in HaCaT cells. The values are expressed as the means ± SE and represent data from three separate studies (n = 3). Error bars with distinct lettering indicate significant differences (p < 0.05). *p < 0.05, **p < 0.01, ***p < 0.001, ns>0.05.

ROS generation is a crucial component of acute inflammatory response. We investigated whether TE directly induces ROS formation. Flow cytometry and immunofluorescence analyses revealed that TE elevated ROS levels in HaCaT cells in a dose-dependent manner, indicating an interaction between cellular oxidation (**Figures 2A, B**). Moreover, the levels of oxidative stress indices in mouse blood, MDA, and ROS increased significantly at 1–2 h and stabilized at 24 h (**Figure 2C**). In addition, TE can rapidly stimulate HaCaT cells to produce a large amount of ROS and significantly increase lipid peroxidation products, mirroring the secretion level of the inflammatory factors mentioned above (**Figure 2D**).

### 3.3 MAPK signaling pathways are required for the TE-induced inflammation

**Figure 3:**
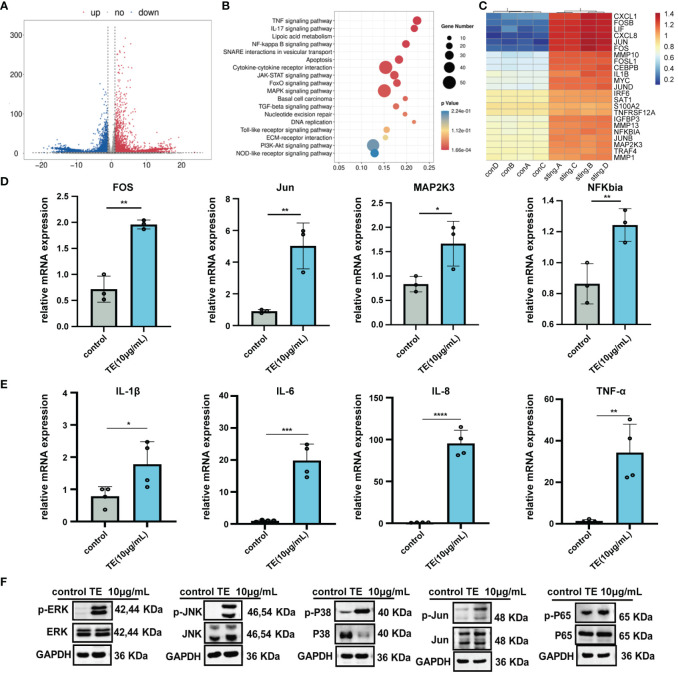
The inflammatory effect of TE on HaCaT cells is associated with MAPK and NF-kB signaling pathways. **(A)** A volcano diagram showing genes with significantly varying expression levels (DEGs). Red plots indicate downregulated genes, while the blue plots indicate upregulated genes. **(B)** KEGG assessment of DEGs. **(C)** Heatmap of DEGs associated with the MAPK and NF-kappaB signaling pathways. **(D)** The MAPK and NF-kappaB signaling pathways’ crosstalk DEGs’ mRNA expression. ***p < 0.001, ****p < 0.0001, **p < 0.01, *p < 0.05. **(E)** Relative mRNA expression levels downstream of MAPK and NF-kappaB signaling pathway. **(F)** Expression levels of the MAPK and NF-kappaB signaling-related proteins. Outcomes are presented as the average ± standard error of at least three separate tests.

To analyze the underlying molecular mechanism of TE in skin inflammation, we performed RNA sequencing to identify altered gene expression. A dependable differential expression (|log2FC|≥1 & q <0.05) of 13,501 mRNAs was found, comprising 642 down-regulated and 953 upregulated genes (**Figure 3A**). Additionally, KEGG analysis revealed that the top nine enriched signaling pathways (P < 0.05) were primarily linked to cell signal transduction and apoptosis progression. These pathways include the MAPK, TNF-a and p53 signaling pathways (**Figure 3B**). Several genes exhibiting notable variations in expression levels were replicated within the TNF-a and MAPK signaling pathways (**Figure 3C**). To validate the RNA sequencing findings, four genes that are abundant in the MAPK pathway were selected from the heatmap. The trends observed for Jun, c-Fos, MA_2_Pk_3_, and NFKBIA were consistent with the RNA-sequencing results (**Figure 3D**). Notably, downstream inflammatory markers IL-6, IL-8, IL-1β, and TNF-α also increased. These findings are consistent with those of earlier *in vivo* and *in vitro* studies of TE inflammation (**Figure 3E**). To verify whether TE-stimulated inflammation is also significantly influenced by the upregulated MAPK genes and NF-kappaB pathways, HaCaT cells were treated with TE (10 μg/mL) dissolved in serum-free medium. Western blotting analysis revealed significant upregulation of phosphorylated p38-MAPK, extracellular signal-regulated kinase (ERK), c-Jun N-terminal kinases (JNK), and NF-kappaB p65 mediators, suggesting that TE activates the NF-kappaB and MAPK pathways, leading to significant increases inflammatory cells (**Figure 3F**). The results *in vivo* further verified that TE can significantly acute inflammation responses (**Supplementary Figure S4**).

### 3.4 Troxerutin suppresses acute inflammatory responses induced by TE

**Figure 4:**
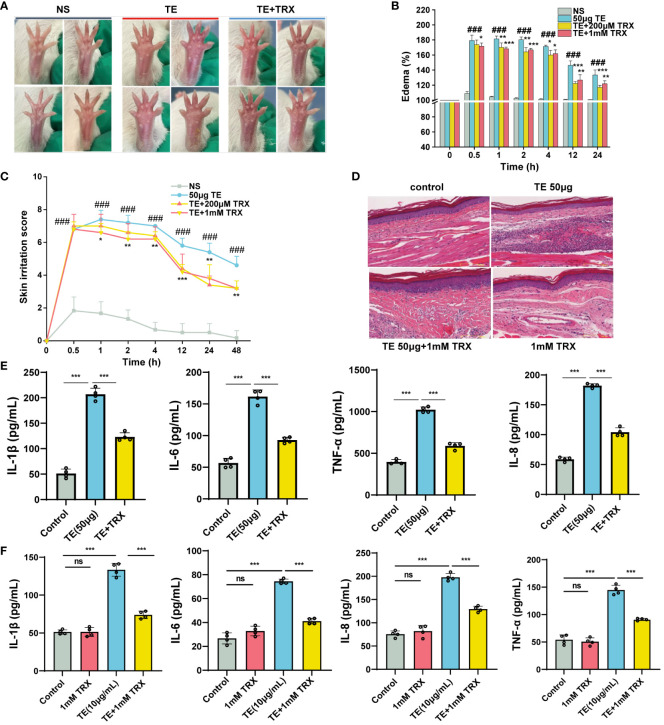
Inhibition of troxerutin (TRX) on the toxicity effect and inflammatory response induced by TE *in vivo* and *in vitro*. **(A)** Changes in toxicity effect with TE (50 μM) combined with TRX (200 μM and 1 mM) at different times in mouse paw. **(B)** Skin swelling percentage and **(C)** irritation score in mice paw. **(D)** Representative pathological section of dermal toxicity (scale bars =100 μm) **(E)** Protein expression levels of four inflammatory cytokines IL-1β, IL-6, IL-8, and TNF-α in the blood of mice that act with TE combined with TRX (1 mM) and **(F)** in HaCaT cells with TRX (1 mM). n = 4 experiments/group. A total of three independent determinations (mean ± SE) supported the findings. A significant difference is shown by error bars with distinct letters (p < 0.05). *p < 0.05, **p < 0.01, ***p < 0.001, ### p < 0.001, ns>0.05.

Troxerutin (TRX) may be beneficial in suppressing inflammation and oxidative stress diseases (19–25). To examine the inhibitory effect of TE stimulation, mouse paws were injected with or without a TRX mixture (0 μm, 200 μm, 1 mM), which had been systemically pretreated with TRX and TE. As expected, TRX treatment successfully reduced the swelling and edema (**Figure 4A**). TRX, at 1 mM, effectively ameliorated plaque and edema symptoms. A significant decline in the percentage of paw swelling was observed within 12–24 h (**Figure 4B**). Skin irritation scores markedly declined after 4 h. However, no discernible dose-dependent inhibitory effect was observed on edema in mice (**Figure 4C**). A histological phenomenon where a reduction in the acute inflammatory pathological alterations in the epidermis was observed compared with that in the TE group after TRX treatment for 12 h (**Figure 4D**). Additionally, TRX significantly reduced the inflammatory response induced by TE. Varying degrees of reduction in the levels of inflammatory cytokines, such as TNF-α, IL-1β, IL-6, and IL-8, were observed in the mice’s blood compared with those in the venom group (**Figure 4E**). Furthermore, HaCaT cells survival increased with TRX treatment at 20, 50, 100, 200, 500, 1000 μM concentrations according to a dose-dependent pattern, especially at a concentration of TRX above 50 μM (**Supplementary Figure S2**). Notably, 1mM TRX alone has no obvious cytotoxicity and also can improve cell viability. Similarly, the levels of inflammatory factors in cells decreased after TRX treatment (**Figure 4F**). These results confirmed TRX’s anti-inflammatory efficacy on TE-induced inflammation.

### 3.5 Inhibition of TRX on TE by inhibiting MAPK signaling pathways and activating Nrf2 signaling pathway

**Figure 5:**
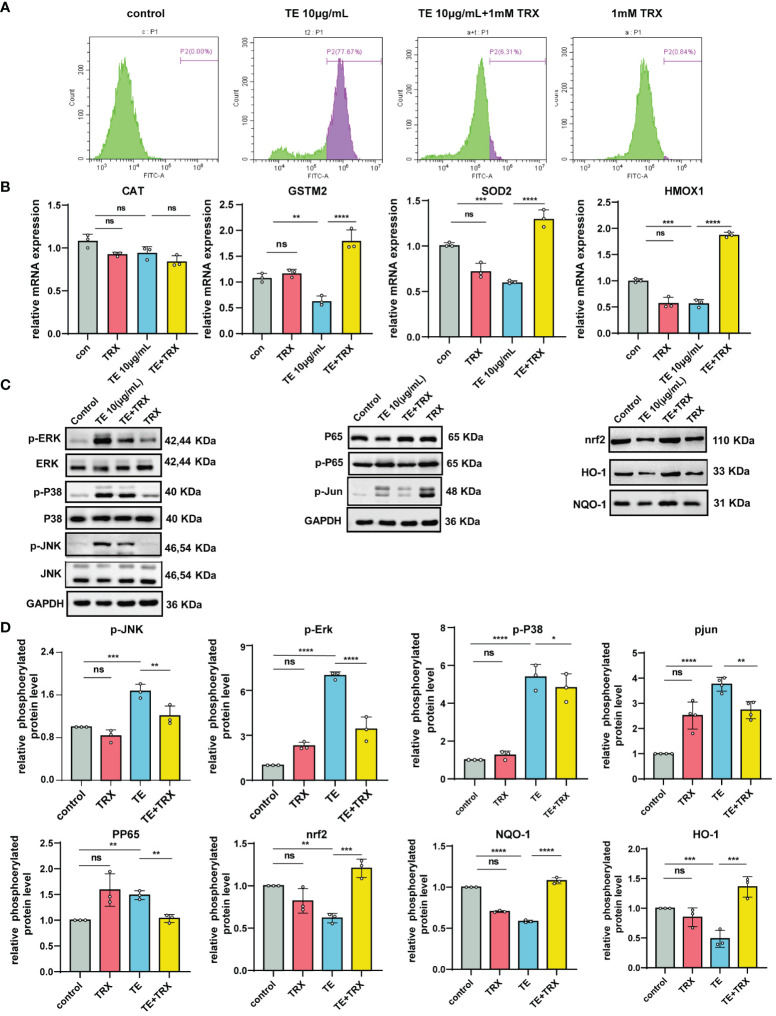
Protective effects of TRX on intracellular ROS generation and inhibition of MAPK and NF-κB by acting nrf2 pathways in TE-stimulated HaCaT cell. **(A)** Production of ROS within cells and measurement of ROS levels via TRX and TE mixed flow cytometer. **(B)** TRX’s impact on the mRNA expression of antioxidant indicators in HaCaT cells that act with TE and TRX mixture. n = 3 experiments/group. **(C)** Expression levels of the protein MAPKs, NF-kappaB, and transcription factor (Nrf2) pathway in HaCaT cell. **(D)** Densitometry measurements for the protein molecular mediators. All outcomes are presented as the average ± standard error of at least three separate studies. *p < 0.05, **p < 0.01, ***p < 0.0001, ****p < 0.001, ns>0.05.

We evaluated the inhibitory effects of TRX on TE-induced oxidative damage and inflammation. The flow cytometry results suggested that intracellular ROS levels decreased in a dose-dependent manner following TRX treatment (**Figure 5A**). Additionally, when TE was incubated with HaCat cells, the relative mRNA expression levels of the antioxidant enzymes, including glutathione S-transferase Mu 2 (GSTM2), heme oxygenase-1 (HMOX1), and superoxide Dismutase 2 (SOD2), decreased compared with those in control, whereas the mRNA expression levels significantly increased when TRX was administered. Notably, the expression of catalase (CAT) was unaffected by TE or TRX. This finding highlights the possibility of using TRX to greatly reduce the oxidative damage caused by TE (**Figure 5B**). Our research has shown that MAPK signaling pathways are essential for TE-induced cytotoxicity, which results in cell inflammatory responses and death. Therefore, we evaluated whether the impact of TRX stimulation blocked the upstream associated with MAPK genes and NF-kappaB signaling pathway in TE-stimulated inflammation (**Figure 5C**). We found that TRX markedly suppressed the phosphorylated levels of ERK1/2, JNK, Jun and NF-kappaB p65, whereas the level of phosphorylated protein p38 was slightly decreased. Furthermore, prior research has demonstrated that TRX prevents oxidative damage by triggering the Nrf2 signaling pathway, a crucial regulator of the antioxidant response within cells that governs the activation of genes that mitigate oxidative stress (30). We hypothesized a similar inhibitory effect of TRX on TE-mediated dermatitis. As expected, the findings revealed that the protein expression levels of transcription factor (Nrf2), heme oxygenase-1 (HO-1), and NAD(P)H quinone oxidoreductase (NQO1) were downregulated when the cells were stimulated with TE but were dramatically elevated after TRX therapy. Based on our results, we postulate that TRX likely binds to certain nrf2 pathway molecule binding sites, which promote HO-1 and NQO1 expression. Furthermore, important genes related to the MAPK pathway were blocked to decrease tissue and HaCaT cell oxidative stress and inflammation (**Figure 5D**).

## 4 Discussion

The skin, the first organ to be exposed to jellyfish venom, has sufficient integrity and maintains resistance to external chemicals by maintaining moisture. Most patients have jellyfish dermatitis, a condition characterized by mild to moderate skin lesions (2–4). Jellyfish venom has been shown to demonstrate several biological actions, such as hemolytic activity, myotoxicity, and cutaneous toxicity, as well as the molecular processes underlying venom-induced cutaneous inflammatory reactions. However, the effectiveness of this treatment is still restricted (5, 6). Inflammatory responses accompanying jellyfish stings are the most common pathophysiological phenomena in jellyfish dermatitis. In our study, TE was injected into mouse paws, and leukocyte infiltration triggered an inflammatory response that resulted in tissue swelling, ischemia, reduced blood flow, and increased tissue damage, which is consistent with the findings from earlier study results (31). Furthermore, cytokines regulate a complex network of connections and have been identified as important modulators of acute inflammatory reactions. HaCaT cells, with different concentrations of TE, can trigger inflammatory factors (IL-1β, IL-6, IL-8, and TNF-α) that rapidly reach their highest level in 2 h. Mice blood cytokine protein expression exhibits a dose-dependent trend of increase. Consistent with these results, TE rapidly and strongly triggered an acute inflammatory response (6, 18, 32). In addition, the accumulation and excessive production of ROS in cells results in oxidative stress, which is one of the primary factors reason for inflammatory diseases. Under homeostatic conditions, a dynamic equilibrium of mechanisms that generate and remove ROS maintains the redox state of cells (33). When mice received Nemopilema nomurai, the levels of MDA and glutathione (GSH) significantly increased (18). Our fluorescence microscopy analysis further indicated that TE dose-dependently induced a significant increase in intercellular ROS and MDA accumulation, which aggravated the lipid peroxidation response. These findings suggest that TE causes inflammation and oxidative stress, leading to severe clinical symptoms.

To understand the relationship between the cytotoxicity of TE and the mechanisms of inflammatory mediator signaling and oxidative damage to the skin, we confirmed the function of the MAPK pathway in the regulation of gene expression. As a group of serine-threonine protein kinases, the MAPK pathway comprises three signaling pathways: ERK, p38 MAPK, and JNK. These pathways are involved in various pathophysiological processes, including cell differentiation, particularly in diseases linked to inflammation (34). Further, decreasing the phosphorylation of MAPK mediators is an important factor in ameliorating inflammatory responses (35). In addition, the MAPK signaling pathway mediates phosphorylation events that activate several transcription factors linked to inflammation, which is related to the NF-kappaB pathway (36, 37). ROS overproduction-induced cytotoxicity is regulated by the release of cytokines as a result of the translocation of nuclear factor NF-kappaB p65 (38). According to recent studies, TE dramatically enhances oxidative stress in mouse skin tissues by regulating the production of proteins linked to the NF-κB and MAPK pathways (18). This result was further supported by our transcriptome data involving TE-stimulated HaCaT cells, revealing the underlying molecular mechanisms of jellyfish dermatitis. We confirmed that phosphor-P38, extracellular ERK, JNK, nuclear factor Jun, and NF-kappaB p65 markedly increased protein levels following exposure to TE. Accordingly, we hypothesized that pro-inflammatory cytokine expression, including TNF-α and IL-6, could be regulated by ERK1/2, JNK, and p38 MAPK phosphorylation upstream of the MAPK pathway. Notably, studies have indicated that ERK is necessary to increase cell viability, whereas JNK and p38-MAPK are linked to many oxidative stress responses that lead to apoptosis (39, 40). Notably, in many inflammatory skin pathogens, JNK activation is a characteristic response to stressful stimuli (40, 41). Our results showed a direct interaction between TE and the expression of major proteins of the MAPK pathway. Further, phosphorylated P38 MAPK and JNK trigger transcription factor Jun and NF-kappaB P65 phosphorylation, leading to a release of substantial downstream inflammatory factors to aggravate pathological phenomena.

TRX has anti-inflammatory and antioxidant effects on inflammatory damage owing to its high water solubility (20). The chemical processes underlying the ability of TRX to protect the skin from venom have not yet been described. Thus, we explored whether TRX can relieve the symptoms associated with jellyfish dermatitis based on a previous mouse dermal model of jellyfish dermatitis. The data showed that TRX significantly inhibited the development of TE-induced dermatitis, with a significant reduction in tissue edema. The pathological results demonstrated a significant reduction in the level of inflammation and elimination of edema. In addition, the cell survival rate can be improved by TRX, which has no harmful effects *in vitro* (42) and effectively suppresses the levels of pro-inflammatory cytokines. Furthermore, intercellular ROS accumulation in TE cells was reduced by TRX treatment. Several major antioxidant enzyme activities, such as those of SOD2, GSTM2, and HMXO-1, can be enhanced by TRX application, whereas the mRNA expression of CAT was not influenced by TE or the TRX mixture, which suggests that TE acts for a short time so that the antioxidant enzymes cannot clear the cytotoxin in time. These results were supported by a previous study (43) showing that TRX exhibits strong free radical scavenging activity and reduction capacity, which may be responsible for its anti-inflammatory effects. Despite mounting evidence that MAPK affects oxidative damage and production, TRX decreases the inflammatory response by decreasing the levels of phosphorylated MAPK mediators (44, 45). We observed that TRX suppressed phosphor-ERK, JNK, p38 MAPK, and NF-kappaB p65 levels. TRX administration can suppress NF-kappaB activation facilitated by MAPK phosphorylation, thereby reducing the degree of toxicity and alleviating inflammation *in vivo* and *in vitro* elicited by the venom from TE.

The cytoprotective cellular mechanism of the Nrf2 signaling pathway activation in HaCaT keratinocytes is widely recognized and has been implicated in the treatment of oxidative stress-related illnesses (35, 46). The Nrf2 signaling pathway plays a crucial role in regulating the transcription of genes encoding endogenous antioxidant enzymes, which are involved in the regulation of cellular redox equilibrium and trigger the transcription of many genes involved in antioxidant defense, such as HO-1 and NQO1 (47–49). In this study, we examined the reduction in Nrf2, HO-1, and NQO1 expression in TE-stimulated HaCaT cells. In contrast, TRX’s antioxidant capacity of TRX dose-dependently increased the Nrf2 signaling pathway activity. These results highlight the ability of TRX to suppress jellyfish dermatitis, thereby enhancing its antioxidative capabilities effectively. Our results showed that the preventive effect of TRX on the inhibition of MAPK pathway activation-induced inflammation was probably mediated by its antioxidative potential against TE. In addition, we hypothesized that TRX may activate a difference in the gene junction site in the MAPK pathway, which suppresses related genes to relieve the inflammatory response.

**Figure 6:**
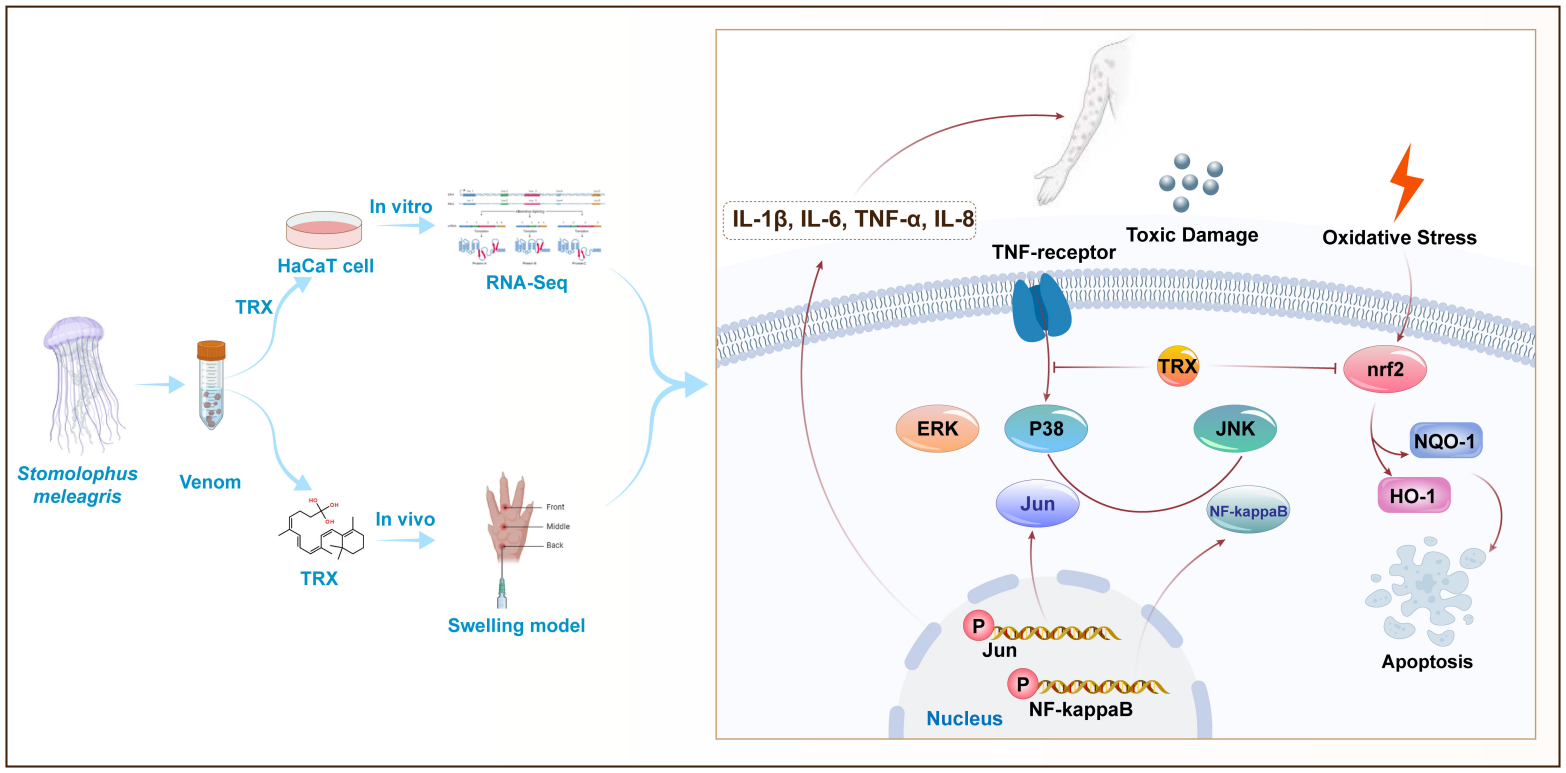
Proposed mechanisms for TRX inhibition in HaCaT cells involve MAPK- NF-kappaB -nrf2 signaling. TE can trigger the cascade phosphorylation of ERK, JNK and P38 upstream of MAPK signaling pathway cells by binding to TNF-αmembrane receptors, further activating nuclear transcription factors Jun and NF-kappaB and releasing a large amount of downstream inflammatory factors IL-1β、IL-6、TNF-αand IL-8. TRX can further inhibit the phosphorylation levels of intracellular Jun and NF-kappaB by inhibited P38, ERK and JNK, eventually attenuating TE-mediated inflammatory responses. In addition, TRX inhibits the TE-mediated nuclear transcription factor nrf2, as well as its attachment proteins NQO-1 and HO-1.

In conclusion, our finding revealed that multiple pathways are involved in the TE induced inflammatory response and oxidative stress in the skin, including the physiological significance of MAPK and NF-kappaB activation. In addition, our results also demonstrate TRX exhibited superior inhibitory potential against inflammatory responses and oxidative stress and upregulated Nrf2 expression by inhibiting the phosphorylation of MAPK and NF-kappaB signaling pathways (**Figure 6**). Moreover, investigations are required to identify the major molecule mechanism(s) ensuring the inhibited effects of TRX, offering valuable insights for research and practical clinical applications.

## Data availability statement

The original contributions presented in the study are included in the article/**Supplementary Material**, further inquiries can be directed to the corresponding authors.

## Ethics statement

The animal studies were approved by Shanghai Changhai Hospital Committee on the Ethics of Animal Experiments at the Chinese Naval Medical University. The studies were conducted in accordance with the local legislation and institutional requirements. Written informed consent was obtained from the owners for the participation of their animals in this study.

## Author contributions

RL: Conceptualization, Formal analysis, Project administration, Validation, Writing – original draft. YW: Conceptualization, Formal analysis, Project administration, Validation, Writing – original draft. WK: Data curation, Investigation, Project administration, Validation, Writing – original draft. WL: Investigation, Writing – review & editing. ZW: Investigation, Writing – review & editing. LX: Project administration, Supervision, Writing – review & editing. JW: Funding acquisition, Project administration, Supervision, Writing – review & editing.
