# Supplementary material for: Troxerutin suppress inflammation response and oxidative stress in jellyfish dermatitis by activating Nrf2/HO-1 signaling pathway
Source: Front Immunol. 2024 May 8;15:1369849. doi: 10.3389/fimmu.2024.1369849 (PMC11109374; doi:10.3389/fimmu.2024.1369849)
Supplement: Supplementary file 1 [file DataSheet_1.docx]

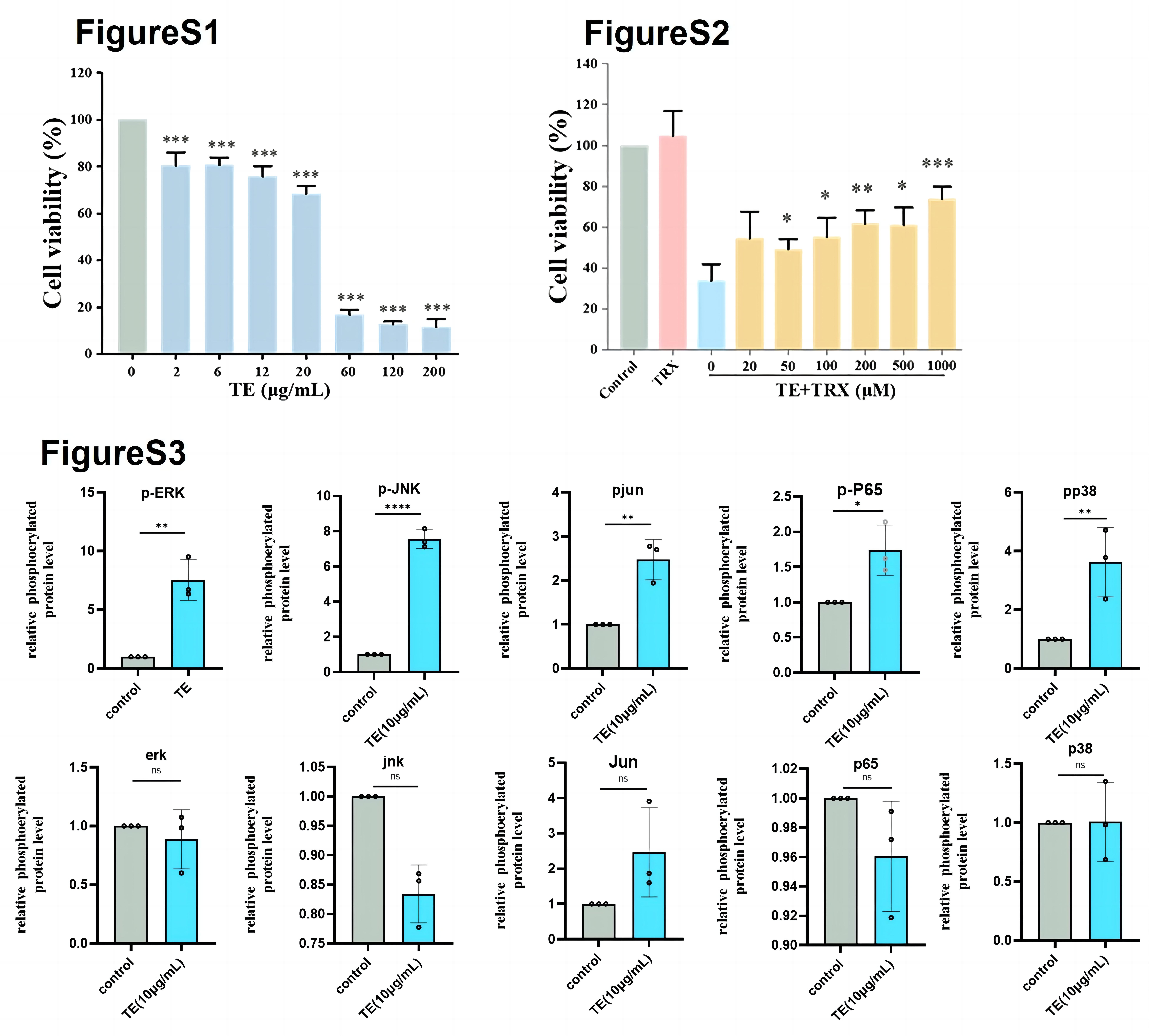


**Table 1：The primer sequences amplified used for RT-PCR.**

| Gene | Sequences |
| --- | --- |
| IL1B-forward | 5′ -TACCTGTCCTGCGTGTTGAAA-3′ |
| IL1B-reverse | 5′ -GGTGCTGATGTACCAGTTGGG -3′ |
| IL6-forward | 5′ -GGTGTTGCCTGCTGCCTTCC-3′ |
| IL6-reverse | 5′ -GTTCTGAAGAGGTGAGTGGCTGTC-3′ |
| IL-8-forward | 5′ -CAGTTTTGCCAAGGAGTGCTAA-3′ |
| IL-8-reverse | 5′ -AAACTTCTCCACAACCCTCTGC-3′ |
| Jun-forward | 5′ -AAGAACTCGGACCTCCTCACCTC-3′ |
| Jun-reverse | 5′ -GCCCGTTGCTGGACTGGATTATC-3′ |
| MAP2K3-forward | 5′ -TCCCAGCCGACCGTTTCTCC-3′ |
| MAP2K3-reverse | 5′ -TCCCGCCCATACTCACCATCAG-3′ |
| NFKbia-forward | 5′ -AGGAGTACGAGCAGATGGTCAAGG-3′ |
| NFKbia-reverse | 5′ -GGCCAAGTGCAGGAACGAGTC-3′ |
| FOS -forward | 5′ -CGTCTTCCTTCGTCTTCACCTACC-3′ |
| FOS-reverse | 5′ -TCATTGCTGCTGCTGCCCTTG-3′ |
| Catalase -forward | 5′ -TTACTCAGGTGCGGGCATTCTA -3′ |
| Catalase-reverse | 5′ -GGATGTGGCTCCCGTAGTCA -3′ |
| GSTM2-forward | 5′ -GCCAAACTCTGCTATGACCCA -3′ |
| GSTM2-reverse | 5′ -TCTCCAAGCCCTCAAATCGG -3′ |
| HMOX1 -forward | 5′ - GTCAGGCAGAGGGTGATAGAAGAG-3′ |
| HMOX1 - reverse | 5′ - AGTGTAAGGACCCATCGGAGAAG -3′ |
| SOD2 - forward | 5′ - GGGACACTTACAAATTGCTGCTT -3′ |
| SOD2 - reverse | 5′ - CATTCTCCCAGTTGATTACATTCC -3′ |
| GAPDH-forward | 5′ - GGAAGCTTGTCATCAATGGAAATC -3′ |
| GAPDH-reverse | 5′ - TGATGACCCTTTTGGCTCCC -3′ |

**Figure S1:** The evaluation of cytotoxicity in HacaT cells treated with S.meleagris venom concentration of (0, 2, 6, 12, 20, 60, 120, 200μg/mL), **Figure S2:** The therapeutic potential of TRX (20,50,100,200,500,1000μM) on cytotoxicity in HacaT cells treated with S.meleagris venom, **Figure S3:** Expression levels of the MAPK and NF-kappaB signaling-related proteins of Western Blot between control group and S.meleagris venom.All results are represented as the mean ± SEM for at least three independent experiments. * *p* < 0.05, ***p* < 0.01, *** *p* < 0.001, **** *p* < 0.0001. **Figure S4**:**Table S1**: PCR primer sequences.
