# Supplementary material for: Troxerutin suppress inflammation response and oxidative stress in jellyfish dermatitis by activating Nrf2/HO-1 signaling pathway
Source: Front Immunol. 2024 May 8;15:1369849. doi: 10.3389/fimmu.2024.1369849 (PMC11109374; doi:10.3389/fimmu.2024.1369849)
Supplement: Supplementary file 2 [file DataSheet_2.pdf]

Figure S4

Expression levels of the MAPK and NF-kappaB signaling-related proteins in vivo

Repeat1:

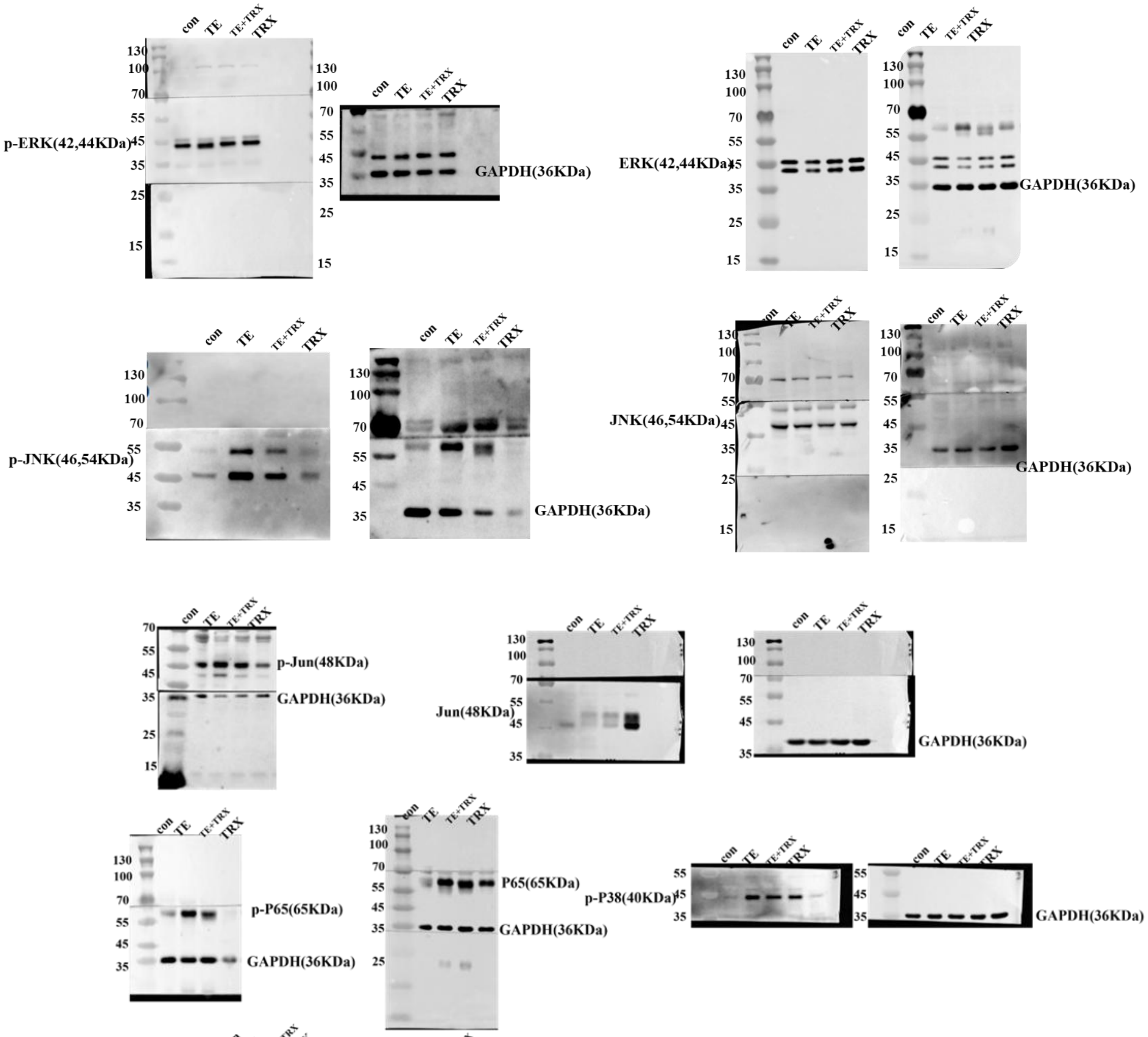

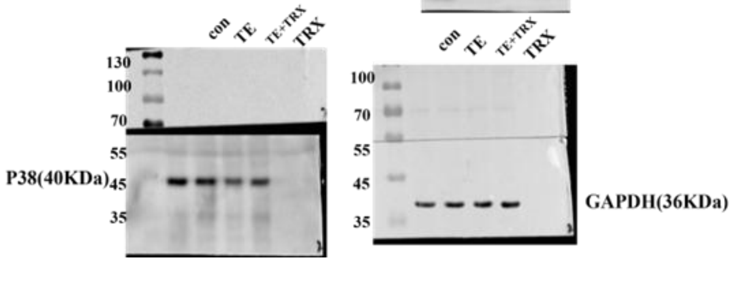

Repeat2:

Repeat2:

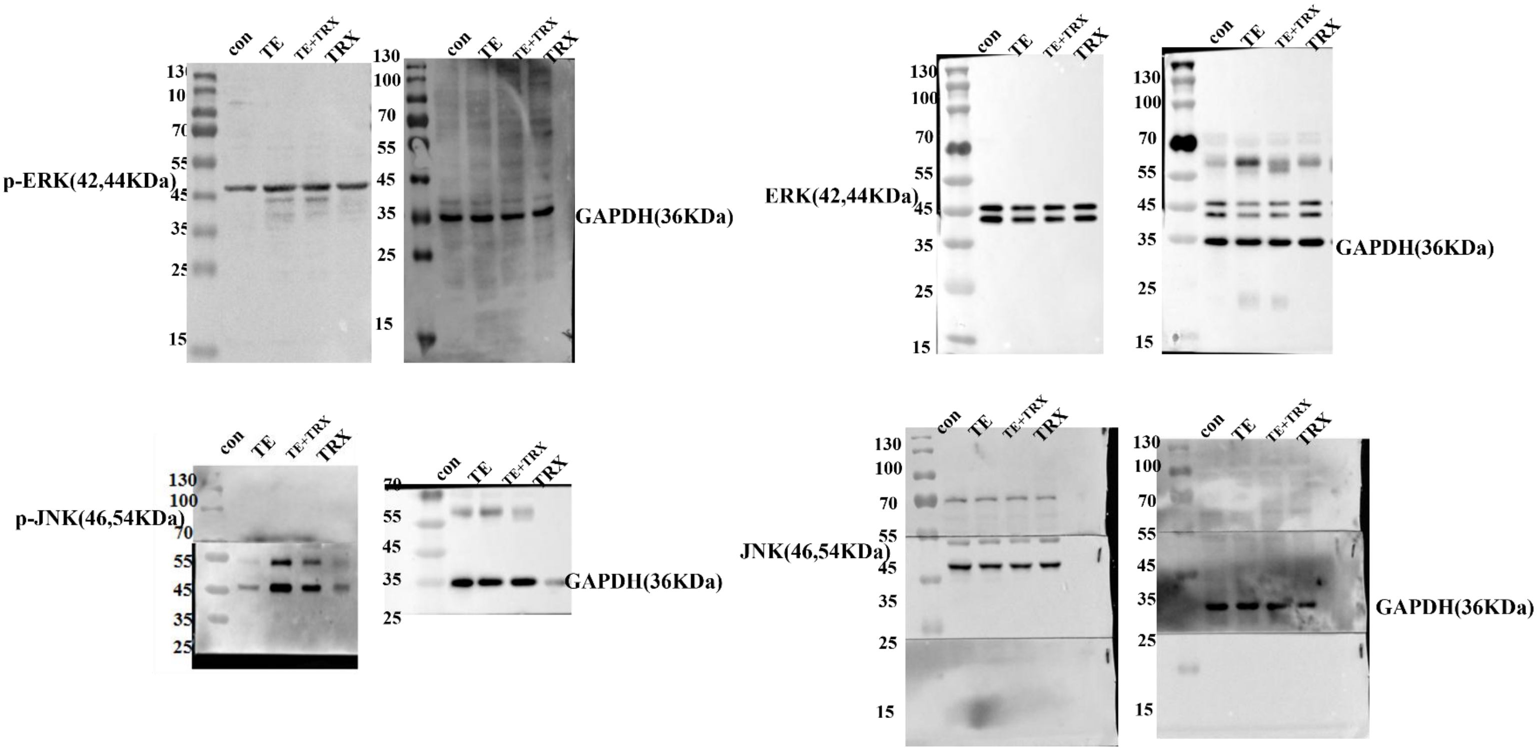
con
TE
TE+TRX
TRX
p-ERK(42,44KDa)
GAPDH(36KDa)
ERK(42,44KDa)
GAPDH(36KDa)
p-JNK(46,54KDa)
GAPDH(36KDa)
JNK(46,54KDa)
GAPDH(36KDa)
130
100
70
55
45
35
25
15

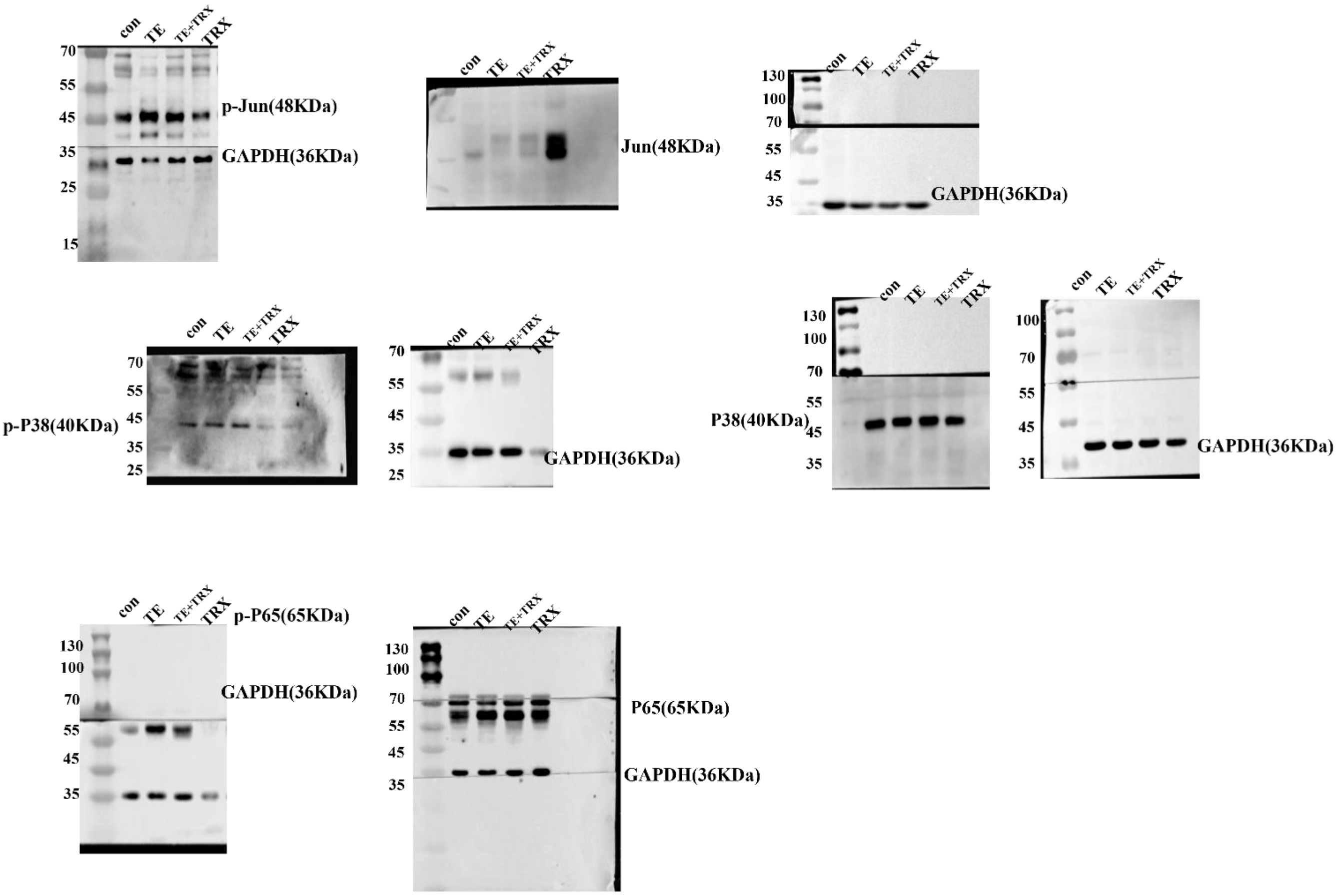
con
TE
TE+TRX
TRX
p-Jun(48KDa)
GAPDH(36KDa)
Jun(48KDa)
GAPDH(36KDa)
p-P38(40KDa)
GAPDH(36KDa)
P38(40KDa)
GAPDH(36KDa)
p-P65(65KDa)
GAPDH(36KDa)
P65(65KDa)
GAPDH(36KDa)
130
100
70
55
45
35
25
15

Repeat3:

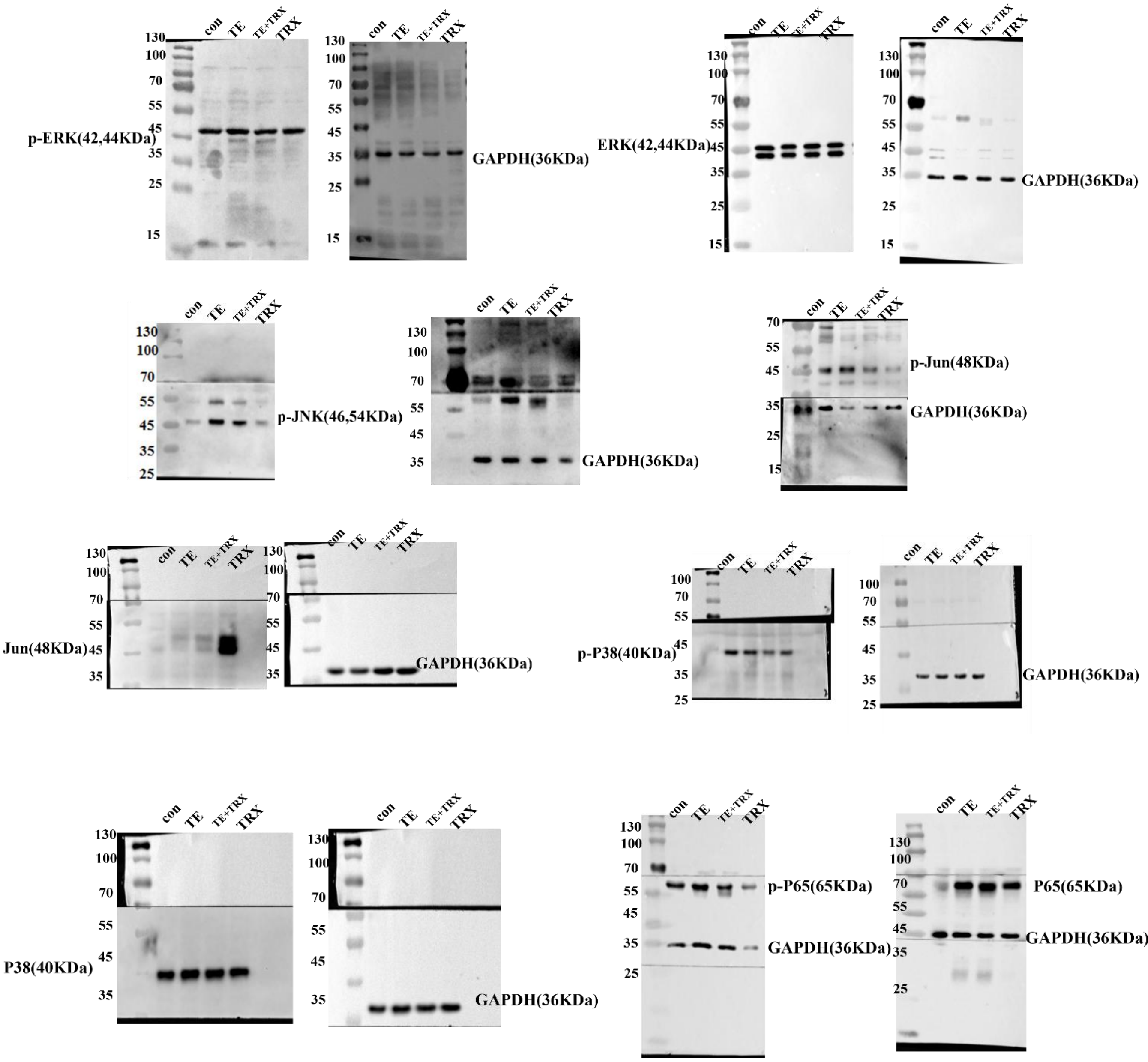

con
TE
TE+TRX
TRX
p-ERK(42,44KDa)
GAPDH(36KDa)
ERK(42,44KDa)
GAPDH(36KDa)
p-JNK(46,54KDa)
GAPDH(36KDa)
p-Jun(48KDa)
GAPDH(36KDa)
Jun(48KDa)
GAPDH(36KDa)
p-P38(40KDa)
GAPDH(36KDa)
P38(40KDa)
GAPDH(36KDa)
p-P65(65KDa)
GAPDH(36KDa)
P65(65KDa)
GAPDH(36KDa)
